# Cerebral Near-Infrared Spectroscopy: A Viable Tool in an Anesthesiologist’s Armamentarium for Pediatric Head and Neck Surgeries

**DOI:** 10.7759/cureus.53592

**Published:** 2024-02-05

**Authors:** Rajasekar Ramadurai, Banupriya Ravichandrane, Surentharraj Elangobaalan, Tamilarasan A Palanisamy

**Affiliations:** 1 Department of Anesthesiology and Critical Care, Jawaharlal Institute of Postgraduate Medical Education and Research, Puducherry, IND

**Keywords:** regional cerebral oxygen saturation (rso2), cerebral perfusion, head and neck surgery, carotid endarterectomy, pediatric anesthesia, near‐infrared spectroscopy

## Abstract

Near-infrared spectroscopy (NIRS) is a noninvasive monitor used regularly in pediatric cardiac surgeries to monitor regional cerebral oxygenation (rScO2). A significant intraoperative cerebral desaturation (>20% from baseline) has been reported with poor neurological outcomes. We describe a case of a six-year-old child with carotid sheath neuroblastoma, located at the carotid bifurcation posted for tumor excision. Intraoperative NIRS monitoring revealed only a transient and insignificant (<10%) fall in the rScO2 during the tumor manipulation, ensuring uninterrupted cerebral circulation. The pediatric population is vulnerable to various physiological changes during anesthesia and surgery, and conserving cerebral function is one of the major goals. Though NIRS has been researched in various surgical specialties, future emphasis must be laid on its use in pediatric head and neck surgeries as a surrogate for cerebral perfusion.

## Introduction

Near-infrared spectroscopy (NIRS) is a portable and noninvasive monitoring tool that provides real‐time information regarding tissue oxygenation. It is regularly used to monitor regional cerebral oxygenation (rScO2) during pediatric cardiac surgeries and neonatal units. Still, its utility in noncardiac surgeries such as head and neck oncological surgeries remains unearthed [1]. Routine cerebral oxygenation is not instigated in all surgeries; however, cerebral desaturation of >20% from baseline correlates to poor neurological outcomes [2]. By providing the anesthesiologists with early indicators of impaired brain perfusion, cerebral NIRS allows for prompt interventions to avert further neurological insults. We report a case of cervical neuroblastoma in a six-year-old female child located in the carotid space where NIRS was employed to monitor rScO2 and guide in the surgical excision of the tumor.

## Case presentation

A six-year-old female child, weighing 16 kg, came with the presenting complaints of swelling in the right side of the neck detected incidentally a year ago. She had no pain or other compressive symptoms. On radiographic evaluation, a well-defined lesion was detected in the right carotid space, which was later diagnosed as neuroblastoma based on biopsy. Following a short course of chemotherapy, she was planned for surgical excision of the tumor.

**Figure 1:**
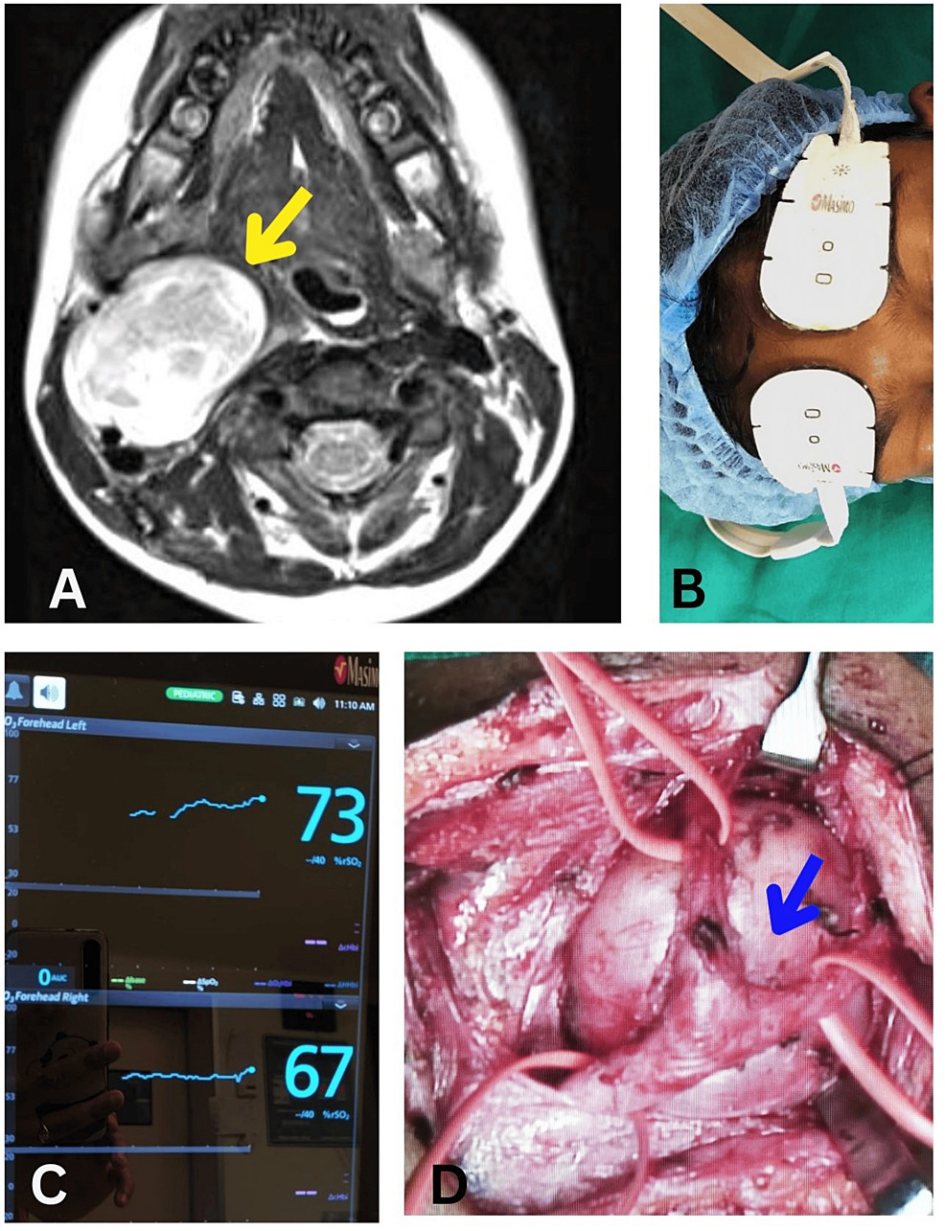
A: MRI displaying the mass in the carotid space (*yellow arrow), splaying the right internal and external carotid arteries). B: NIRS monitoring electrodes attached to the patient. C: Baseline recording of NIRS. D: Surgical location of the tumor (posterior to the bifurcation of the common carotid artery). *blue arrow

On pre-anesthetic evaluation, her baseline vitals and routine investigations were within normal limits. Magnetic resonance imaging (MRI) revealed a 5.5*3.5*3.2 cm mass in the right carotid space, splaying the right internal and external carotid arteries and extending up to the base of the skull without any intracranial extension (Figure 1A). Informed high-risk consent was obtained and adequate blood and blood products were arranged. On the day of the surgery, after ensuring adequate NPO status, the patient was shifted to the operating room, and the American Society of Anesthesiologists (ASA) standard monitors were attached. Prior to the anesthetic induction, NIRS (Root®, Masimo Corporation, Irvine, CA, USA) electrodes were attached to the forehead, and baseline values were obtained: right, 67%; left, 73% (Figure 1B, 1C). The patient was induced through a preexisting intravenous cannula with fentanyl, propofol, and atracurium. The child was intubated on the first attempt using a direct laryngoscopy (glottic view, CL grade 1) with a 5.5 mm internal diameter (ID) cuffed endotracheal tube (ETT). The left radial artery was cannulated for invasive blood pressure monitoring, and a right femoral venous access was established. The child was positioned supine with the head turned to the contralateral side for adequate surgical exposure. Intraoperative analgesia was dealt with intravenous opioids.

**Figure 2:**
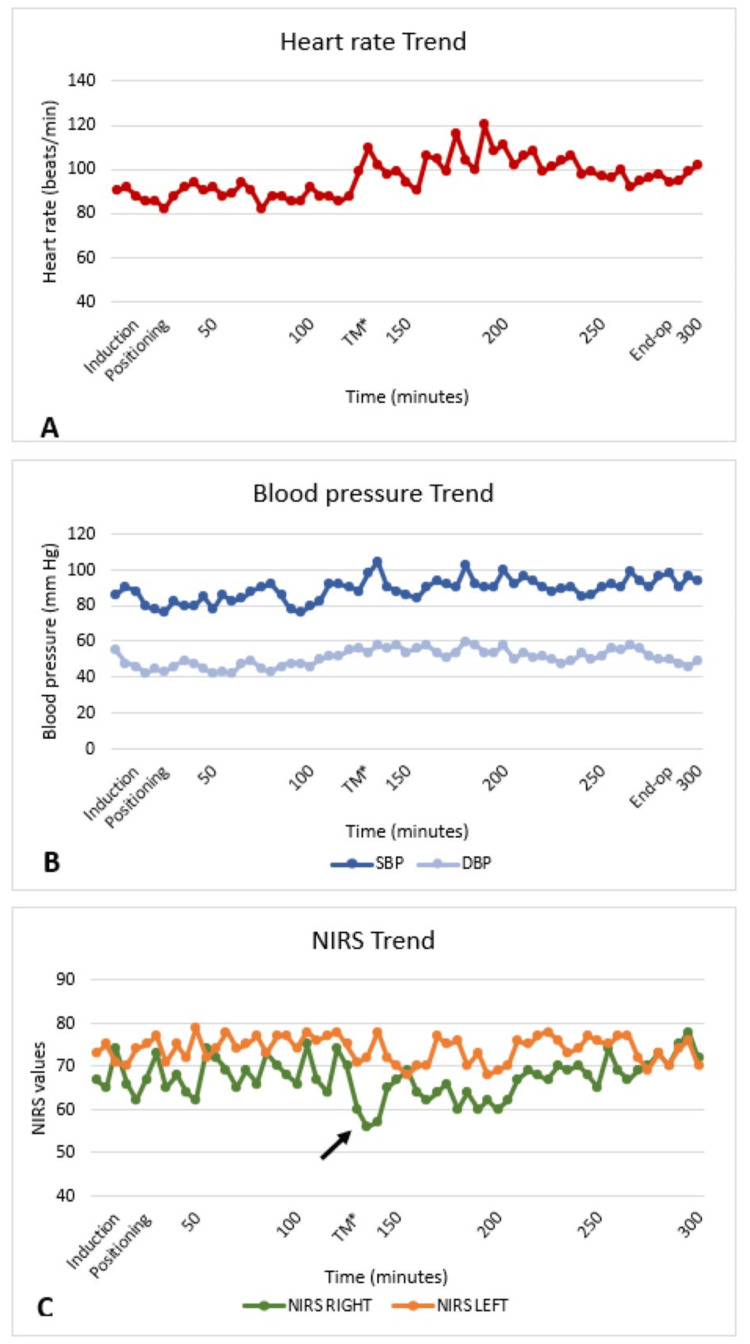
Graphical trend of hemodynamics. A) Heart rate, B) Blood pressure, C) NIRS values Arrow: Drop in NIRS during tumor manipulation; TM: tumor manipulation

 The surgery lasted for seven hours as it required meticulous dissection due to the complex location of the tumor (Figure 1D). Eternal vigilance and regular communication with the surgeon were ensured throughout the procedure. The need for carotid cross-clamping, carotid resection, and inadvertent vascular injury were anticipated, and measures to counter the situation were kept in readiness. However, the intraoperative period was uneventful and did not require major carotid resection. The hemodynamics were stable except for a few episodes of tachycardia during tumor resection which settled with fentanyl boluses. There was a transient fall in the NIRS (<10% from baseline) during the tumor manipulation and carotid handling; however, the NIRS returned to baseline upon releasing the tumor (Figure 2). The malignant mass was removed in toto, and the rest of the operative period was uneventful with minimal blood loss. The patient was extubated after ensuring adequate reversal of neuromuscular blockade once the patient was awake and after ruling out tracheal edema with a leak test. The patient was shifted to the ICU for postoperative care, and the postoperative period was uneventful.

## Discussion

Conservation of cerebral function is one of the major goals of pediatric anesthesia. The pediatric population, especially premature and term neonates, is vulnerable to various physiological changes that occur during anesthesia and surgery [1]. NIRS is being used currently to monitor and detect episodes of cerebral ischemia both intraoperatively and postoperatively for pediatric cardiac surgeries where decreased rScO2 has been associated with adverse neurological outcomes. Its utility in the neonatal and pediatric intensive care units has also evolved, particularly for the management of premature neonates and perinatal hypoxia [2]. However, literature is sparse on the value of cerebral NIRS monitoring during noncardiac surgery except for carotid endarterectomy procedures. Novel applications of NIRS include evaluating tissue oxygenation of noncerebral organs such as liver transplant graft, splanchnic perfusion, and free flaps. Researchers have also recently postulated the combined use of cerebral and somatic NIRS monitoring during anesthesia in pediatric noncardiac surgeries [3].

The main anesthetic goal in carotid surgeries is to optimize cerebral perfusion. Intraoperative neurological monitoring is warranted for early detection of cerebral ischemia, which is usually reversible. Transcranial Doppler (TCD), electroencephalogram (EEG), somatosensory-evoked potential (SSEP), and cerebral oximetry are a few options available. However, the complexity and invasiveness of these monitors preclude their usage. NIRS, a noninvasive, portable, site-specific, and continuous monitoring tool, can be used as a surrogate to monitor cerebral perfusion, with the added advantage of continuing the monitoring in the postoperative period in the ICU.

 In the purview of head and neck surgeries which may require carotid clamping as in our case, NIRS can guide in identifying impaired cerebral circulation early and the need for intraoperative carotid bypass shunting [4]. NIRS monitoring in addition to standard ASA monitors was established in our case for the same reason. It also aided during the dissection in the vicinity of the carotid where a few minor dips in NIRS (<10% from baseline) were observed which gained back to normal on cessation of the surgery. The monitoring was applied before induction of anesthesia to obtain a baseline value as it is recommended to act upon changes from baseline (<20%) and trends in NIRS readings rather than an absolute number [1]. NIRS has been found to detect intraoperative cerebral desaturation in surgeries requiring extensive head rotation or extension [5]. In situations where the collateral carotid flow may be depleted due to external compression or surgical manipulation similar to our setting, NIRS may serve as a guide for the safe positioning of the patients as well. However, we did not notice any such episodes attributable to the neck positioning.

## Conclusions

NIRS can be used as a surrogate to monitor cerebral perfusion in patients undergoing major head and neck and vascular surgeries. Certain physiological changes associated with anesthesia, positioning, and surgery, especially in children, make them more vulnerable, and conserving cerebral function is one of the major goals. Though NIRS has been researched in various surgical specialties, future emphasis must be laid on its use in pediatric head and neck surgeries as a surrogate for cerebral perfusion.

There is a paucity of literature on NIRS in pediatric noncardiac surgeries, and pediatric head and neck surgeries are one subset to ponder upon for future research and to expand the utility of NIRS in anesthesia.
